# Comparison of Multivariate ANOVA-Based Approaches for the Determination of Relevant Variables in Experimentally Designed Metabolomic Studies

**DOI:** 10.3390/molecules27103304

**Published:** 2022-05-20

**Authors:** Miriam Pérez-Cova, Stefan Platikanov, Dwight R. Stoll, Romà Tauler, Joaquim Jaumot

**Affiliations:** 1 Department of Environmental Chemistry, IDAEA-CSIC, Jordi Girona 18-26, E08034 Barcelona, Spain; mpcqam@idaea.csic.es (M.P.-C.); stefan.platikanov@idaea.csic.es (S.P.); rtaqam@idaea.csic.es (R.T.); 2 Department of Chemical Engineering and Analytical Chemistry, University of Barcelona, Diagonal 647, E08028 Barcelona, Spain; 3 Department of Chemistry, Gustavus Adolphus College, 800 West College Avenue, Saint Peter, MN 56082, USA; dstoll@gustavus.edu

**Keywords:** feature detection, ANOVA, ASCA, rMANOVA, GASCA, metabolomics, biomarkers

## Abstract

The use of chemometric methods based on the analysis of variances (ANOVA) allows evaluation of the statistical significance of the experimental factors used in a study. However, classical multivariate ANOVA (MANOVA) has a number of requirements that make it impractical for dealing with metabolomics data. For this reason, in recent years, different options have appeared that overcome these limitations. In this work, we evaluate the performance of three of these multivariate ANOVA-based methods (ANOVA simultaneous component analysis—ASCA, regularized MANOVA–rMANOVA, and Group-wise ANOVA-simultaneous component analysis—GASCA) in the framework of metabolomics studies. Our main goals are to compare these various ANOVA-based approaches and evaluate their performance on experimentally designed metabolomic studies to find the significant factors and identify the most relevant variables (potential markers) from the obtained results. Two experimental data sets were generated employing liquid chromatography coupled to mass spectrometry (LC-MS) with different complexity in the design to evaluate the performance of the statistical approaches. Results show that the three considered ANOVA-based methods have a similar performance in detecting statistically significant factors. However, relevant variables pointed by GASCA seem to be more reliable as there is a strong similarity with those variables detected by the widely used partial least squares discriminant analysis (PLS-DA) method.

## 1. Introduction

In recent years, chemometric tools have been used to analyze omic data and, in particular, metabolomic data obtained mainly through the hyphenation of chromatographic and mass spectrometric techniques [1]. These studies have different goals for which these chemometric tools are helpful, as introduced below [2].

First, chemometric approaches such as classification (or discrimination) methods allow the differentiation of groups of samples (i.e., case and control samples) and, more interestingly, the detection of variables that discriminate between these groups [3]. These variables are usually called markers, and can be associated in metabolomic studies with specific molecules that, for instance, are altered due to a certain exposure or treatment. The selection of these relevant variables that allow the characterization of the different groups of samples (also known as the feature selection step) is critical in the analysis of metabolomic datasets. In fact, the biological interpretation of the metabolic changes observed between sample groups is often based exclusively on the selected variables. A widely used example of these tools is the partial least squares discriminant analysis (PLS-DA) [4], a supervised method (i.e., it uses information about the identity of samples when building up the calibration model) focused on the differences between the sample types [5]. In addition, the use of variable selection methods helps to distinguish between the variables that are the most related to each type of sample and those that have a more significant influence on achieving a correct differentiation [6,7]. The two most used variable selection methods are the Selectivity Ratio (SR) [8] and the Variable Influence on Projection (VIP) scores [9]. However, as highlighted in the literature, the incorrect use of these methods can lead to misleading results because PLS-DA tends to overfit data [10,11]. For this reason, interest in alternative methods for sample discrimination and variable selection is increasing (e.g., principal component analysis in combination with linear discriminant analysis (PCA-LDA) or principal components-discriminant function analysis (PC-DFA)). Nevertheless, most of these alternative methods are non-linear approaches (i.e., machine learning approaches such as neural networks or random forests), allowing both the samples’ discrimination and the determination of the variables that most strongly influence the model [11,12]. On the one hand, these methods can often handle datasets of thousands of variables as well as missing values without pre-processing required. They are also robust to overfitting and outliers. On the other hand, visualization is rather complex and difficult to interpret. The selection of the appropriate parameters is crucial, and a worse classification is encountered when compared with PLS-DA. Additionally, PLS-DA provides a better dimensionality reduction. This problem has already been addressed, and previous works have reported comparisons between different discriminant methods [11].

In contrast, methods focused on evaluating the statistical significance of the studied experimental factors have arisen in the last years. Various approaches have appeared with the common characteristic of relying on ANOVA to decompose the data variance as a function of the experimental design and the considered factors [13]. First, multivariate ANOVA (MANOVA) was proposed [14]. However, its main limitation is primarily related to the required sample size: MANOVA has a strong requirement of having more samples than variables. In metabolomic studies, the most common scenario is to have more variables than samples, which limits the success of this approach [15]. For this reason, alternative approaches were proposed allowing the multivariate data analysis without the need for meeting these strict MANOVA requirements (i.e., sample size and independency, variables multivariate normality, equal group covariance matrices) due to a previous step of data compression. These approaches can be divided into those that perform the data compression step using principal component analysis (PCA, or similar techniques such as Simultaneous Component Analysis, SCA) and those using regression-based methods such as Partial Least Squares (such as ANOVA-PLS [16] or ANOVA Target Projection [17]). Finally, PCA-based approaches seem to be more successful, which has led to the proposal of a variety of methods with this common feature.

The main difference between these PCA-based approaches is how the compression step is implemented to the factor matrices obtained after ANOVA decomposition. The ANOVA-PCA method was initially proposed [18]. In ANOVA-PCA, the residuals were added to the effects’ matrix before their evaluation. Later, ANOVA Simultaneous Component Analysis (ASCA) was presented, with remarkable success [19,20]. The main difference between ASCA and the previous methods is that ASCA does not consider the residuals for modelling the ANOVA-decomposed matrices of the effects. In addition, ASCA assumes both equal variance and no correlation between the considered variables, which could affect the obtained models and hinder their interpretation. Alternative methods such as rMANOVA (regularized MANOVA) have been proposed to overcome these limitations [21]. rMANOVA is a kind of intermediate method with features between MANOVA and ASCA, since it allows the variable correlation without forcing all variance equality. Similarly, the GASCA (group-wise ANOVA-simultaneous component analysis) method has been presented [22]. GASCA attempts to overcome limitations of ASCA by using an approximation based on group-wise sparsity in the presence of correlated variables to facilitate interpretation. These last two methods have been proposed for the analysis of omics data that are characterized by their high dimensionality in the direction of the variables (and a reduced number of samples) and their sparsity due to the presence of a large number of variables that do not present a response for certain samples (i.e., large number of zero elements) [23].

In addition to providing information on the statistical significance of the experimental factors studied, these ANOVA-based methods can also determine the variables most related to the considered experimental factor (i.e., molecules that can be considered markers for the different sample groups). Knowledge related to potential markers can be obtained similarly to that described above when PLS-DA is used. However, only some implementations of these ANOVA-based methods enable this variable selection in a straightforward way, and, in some cases, a reliable determination of potential markers is difficult to achieve [21]. Hence, an in-depth comparison of the performance of the main aforementioned ANOVA-based methods is needed, on the one hand, to evaluate the significant factors of the experimental design, and on the other hand, to assess the identification of the most relevant variables that discriminate sample groups. The ideal scenario would be to find the method that best accomplishes both goals in a single analysis.

In this work, we have evaluated the ability of these ANOVA-based methods to detect the variables responsible for the differences between groups of samples. In this way, the ASCA, rMANOVA and GASCA results are compared, taking as reference the most relevant variables determined by standard methods such as univariate statistical tests and multivariate PLS-DA analysis using VIP Scores as the variable selection method. This study was carried out using experimental datasets of different complexity obtained by liquid chromatography coupled to mass spectrometry (LC-MS). Two experiments were performed: a case with only one factor in the design (yeast samples with two extraction protocols), and a more complex case with multiple factors (zebrafish embryos samples exposed to two endocrine disruptor chemicals (EDCs), each at two concentration levels). In these examples, the effects of the design factors are analyzed both using the chromatograms (total ion current chromatograms) and from the areas of the different analytes (mass values) observed in the data.

## 2. Results

The performance of the different multivariate ANOVA-based methods has been compared considering the two following aspects: the statistical significance of the experimental factors (i.e., lipid extraction on yeast growth dataset and exposure level on zebrafish dataset) and the relevant variables selected for characterization of samples. This list of selected relevant variables was then compared with the results obtained by other widely used approaches (particularly PLS-DA variable selection methods).

### 2.1. Statistical Assessment of Experimental Factors Effects

First, the three ANOVA-based methods were compared using the TIC chromatograms of the yeast samples both in positive and negative ionization modes. In this case, the variables are retention times at which relevant compounds are eluting (e.g., these molecules are presented exclusively in only one sample group, or the peak height is different according to the various sample groups). Since a replicate of the sphingolipid samples was lost (Extraction B), a balanced data set could not be generated (eight samples of Extraction A and seven samples of Extraction B). Thus, when required, one of the samples of Extraction A was removed to allow the study to consider a balanced data set.

Table 1 shows the results obtained for the three multivariate ANOVA-based methods. In most cases, the experimental design factor studied (i.e., lipid extraction on yeast growth dataset and exposure level on zebrafish dataset) could be regarded as statistically significant. In ASCA and rMANOVA, the obtained *p*-values were very close to the lower threshold marked by the number of performed permutations set to 10,000, indicating a large significant effect. In contrast, GASCA results showed some differences between the results obtained for positive or negative MS ionization. The calculated *p*-values for TICs in the positive ionization mode (0.001) were lower than those calculated for the negative ionization mode (0.039). This outcome was reasonable because the lipids extracted in both extractions provided several different signals in the positive mode (due to the variety of families of extracted lipids giving more variability in the measured signal). Still, fewer differences were observed for the negative ionization mode (i.e., fewer lipids provided an observable signal in the MS spectra). If the features matrix is considered, similar results were obtained between the two ionization modes. The ASCA results did not show relevant differences between positive and negative ionization modes, and the *p*-values obtained were similar to those for rMANOVA and GASCA. In this case, all methods detected a clear statistical significance effect for the factor representing the type of extraction (probably due to the major variability in the features matrix over the TICs matrix when considering the values for the chromatograms and the ROI determined areas).

The study of the zebrafish samples allowed for a more in-depth study. Here, the feature matrix contains the areas of the variables filtered after ROI procedure. These variables are expressed as *m*/*z* values and can be associated with metabolites by their accurate mass and their fragmentation pattern (the matches between the experimental and theoretical MS/MS spectra). Regarding the zebrafish dataset, there were two possible comparisons at two dose/concentration levels for each chemical defining the studied experimental factors: control vs. low and control vs. high. In addition, there was also a three-level study considering control vs. low vs. high. In this study, it was observed that the effects caused by BPA or E2 are different. In the case of BPA, almost all comparisons provided significant *p*-values (except the two-level studies evaluated by GASCA). In contrast, for E2, only rMANOVA found a statistically significant effect of the chemical exposure in two-level studies (both low and high doses). We noted that ASCA did not detect the effect of E2 at low concentration as statistically significant, which from a biological point of view makes sense (i.e., E2 is a natural estrogenic hormone, whereas BPA is an exogenous endocrine disruptor [24]). Thus, it could be expected that E2 would have smaller effects compared to BPA. In contrast, rMANOVA found a statistically significant effect even in the case of E2, which seemed to point out it was the more sensitive ANOVA-based multivariate with regards to detecting differences between the considered groups. However, it was not clear if these differences were caused by real potential markers, or could be related to experimental error (e.g., background contributions, badly detected metabolites). This hypothesis could be reinforced when considering the list of potential markers detected by each method (see below) and the significant differences between potential candidates detected by rMANOVA and the PLS-DA VIP scores approach. If the ternary systems were considered, all three methods identified statistically significant effects in the case of BPA, whereas there were divergent results with E2. ASCA and rMANOVA provided statistically significant *p*-values, but GASCA did not determine a ternary effect. These results agreed with what was observed in the individual two-level studies (control vs. low and control vs. high) in which there was no significant effect for GASCA in any case. In contrast, ASCA and rMANOVA gave a significant effect when the control vs. high dose was considered.

From these results, it seemed clear that they did not always provide analogous results, despite some similarities being observed between the three ANOVA-based methods. If we consider ASCA as the reference ANOVA-based method due to its most common use, a direct relationship with rMANOVA or GASCA results cannot be established. It seemed that, in general, rMANOVA tended to determine more statistically significant effects with results similar to ASCA. This behavior could be expected as there is a clear relationship between ASCA and rMANOVA established by the regularization factor (δ). In contrast, GASCA (especially in the case of the feature matrix analysis, probably due to the sparser data structure) did not detect these minor effects and, consequently, the design factor was not identified as statistically significant. For example, in the case of zebrafish samples exposed to low-level E2, no relevant effects were expected from a biological viewpoint. Furthermore, these results were confirmed by the PCA analysis of zebrafish exposed to E2 (see Figure S1 in Supplementary Materials). The PC1 vs. PC2 scores diagram shows that high-dose exposed samples grouped together, far from the control and low-dose samples. In contrast, the control and low-dose samples were much closer and, therefore, were not identified as statistically different.

### 2.2. Impact on Variable Selection

In addition to the previous statistical significance study, applying methods based on the combination of ANOVA and factor analysis allowed the exploration of the distribution of samples and variables in the new dimensional space defined by the principal components. Considering the scores diagrams, in all cases, the ANOVA-based methods enabled differentiation of the samples based on the factor studied, including in cases where the statistical study did not identify statistically significant factors (for example, the exposure to low concentration E2).

**Figure 1:**
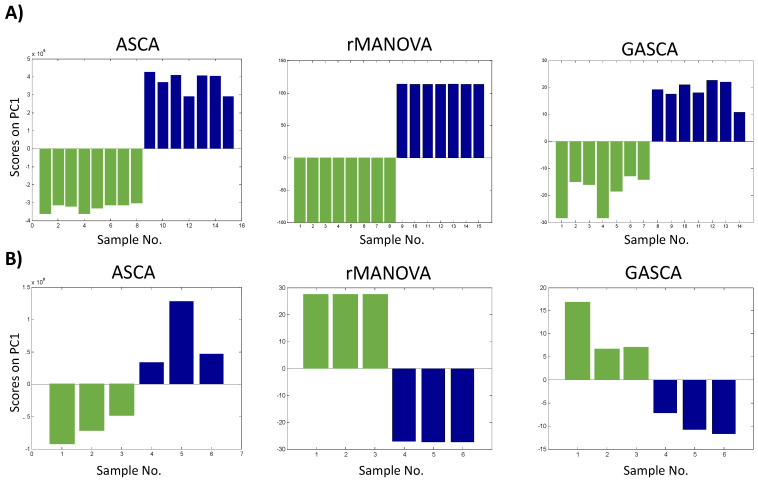
Exploration of the scores generated by the ANOVA-based methods: values of the first component. (**A**) TICs yeast positive. Sample colouring depending on the factor studied: green bars–extraction A: phospholipids, and blue bars—extraction B: sphingolipids; (**B**) Zebrafish embryos exposed to low–dose BPA. Sample colouring depending on the factor studied: green bars—control samples and blue bars—low-dose BPA treatment.

Figure 1 shows as an example the results obtained in two cases. In the first row, the results obtained in the study of the TICs of the yeast experiment in positive mode are shown (the factor was identified as significant in all cases). For the three methods (ASCA, rMANOVA, and GASCA), the first component differentiated by sample type (Figure 1A). The largest within-group difference was observed for GASCA, and to a minor extent for ASCA. In contrast, rMANOVA showed a significant difference among the different types of samples, but almost no differences between the different samples of a particular type. This may reflect the impact of the ANOVA decomposition in the different approaches. This decomposition seemed to force a major similarity within group samples in rMANOVA, whereas ASCA and GASCA could leave more variability.

In the second row of plots (Figure 1B), results for the study of the features matrix of the treatment of fish with low-dose BPA compared with controls are shown. In this case, the effect detected by GASCA was not significant (*p*-value > 0.05) and, although the scores plot discriminated between the control and exposed samples, this difference was minor when compared to rMANOVA, in which the behaviour of the sample types was much more distinct. ASCA showed an intermediate performance giving statistical significance to the factor, but with a representation of the score values similar to GASCA.

**Figure 2:**
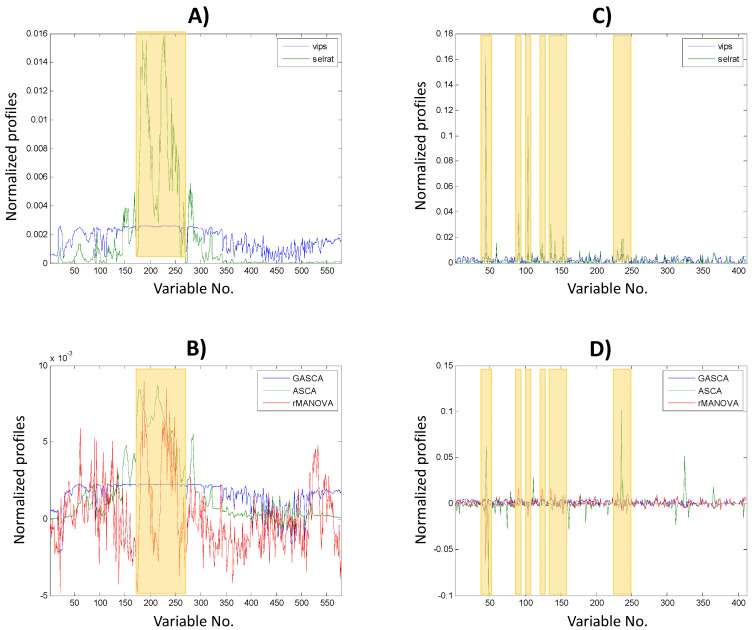
Comparison of the loadings obtained by PLS-DA variable selection methods and ANOVA-based methods. (**A**) TICs yeast positive PLS-DA profiles: VIP scores and selectivity ratio; (**B**) TICs yeast positive ANOVA–based approaches: ASCA, rMANOVA, and GASCA loadings; (**C**) Zebrafish embryos exposed to low–dose BPA PLS-DA profiles: VIP scores and selectivity ratio; (**D**) Zebrafish embryos exposed to low–dose BPA ANOVA–based approaches: ASCA, rMANOVA, and GASCA profiles. In each plot, profiles were normalized to an equal area for representation in the same scale. Shadowed boxes represent regions with a high number of relevant variables.

Figure 2 shows how the variables behave in the cases discussed above (i.e., TICs yeast positive, A and B panels, and zebrafish embryos exposed to low-dose BPA, C, and D panels). In addition to the profiles provided by the ANOVA-based methods and to compare with a widely used method in the field of metabolomics, profiles of the variable selection approaches (e.g., Selectivity Ratio and the VIP Scores) obtained by PLS-DA are also shown. In these PLS-DA models, classes were defined according to the used experimental design. For instance, in the yeast growth studies, samples from extraction A were set as a class (0) whereas samples from extraction B were set as another class (1). Figure 2A shows the profiles obtained by PLS-DA, and Figure 2B shows those obtained by methods based on different multivariate ANOVA methods previously used for the analysis of the TICs yeast study in positive ionization mode. In both cases, variable channels (i.e., retention times of the TIC chromatograms) between 200 and 250 were highlighted as the chromatographic regions enabling differentiation between sample types. Moreover, when considering the first loadings profiles corresponding to the ANOVA-based methods factor decomposed matrices, different patterns could be distinguished. The ASCA loading profile resembled the TIC chromatogram of the sample and the PLS-DA selectivity ratio profile (see similarity between these profiles in Figure 2A,B). Instead, the GASCA loading profile was more similar to the profile obtained for the PLS-DA VIPs scores. Finally, the rMANOVA profile was the most different to the other methods, but positive and negative features were observed in the profiles.

In the case of the features matrices from the ROI analysis for the study of zebrafish embryos with low-dose exposure, the loadings profiles are shown in Figure 2C for the PLS-DA based methods and Figure 2D for the ANOVA-based methods. Similarly, there is an observable link between the variables relevant for both PLS-DA and ANOVA-based methods (i.e., regions that showed larger positive contributions for PLS-DA methods and positive or negative contributions for ASCA methods, as highlighted by the shadowed boxes in each figure). Focusing on the ANOVA-based profiles, the ASCA profile was the most different from the other approaches. A quantitative evaluation of the similarity of the profiles can also be performed by calculating the correlation coefficient between the different sets of profiles (Table S1). For example, in the case of the study of yeast TICs in the positive ionization mode, the ASCA profile was more similar to that obtained by the selectivity ratio approach (0.83), while GASCA was more similar to the profiles obtained using the VIPs scores (0.93). In contrast, rMANOVA was more different to the other profiles (lower coefficient values). Additionally, the same trend was also observed for the rest of the studies when considering the entire Table S1. In general, a good similarity was found between the loading profiles obtained for the different approaches with relatively large correlation coefficients. However, the similarity of the loading profiles resolved for GASCA and the PLS-DA VIP scores could be highlighted because, in all cases, they showed the highest correlation values (all had a value above 0.89).

**Figure 3:**
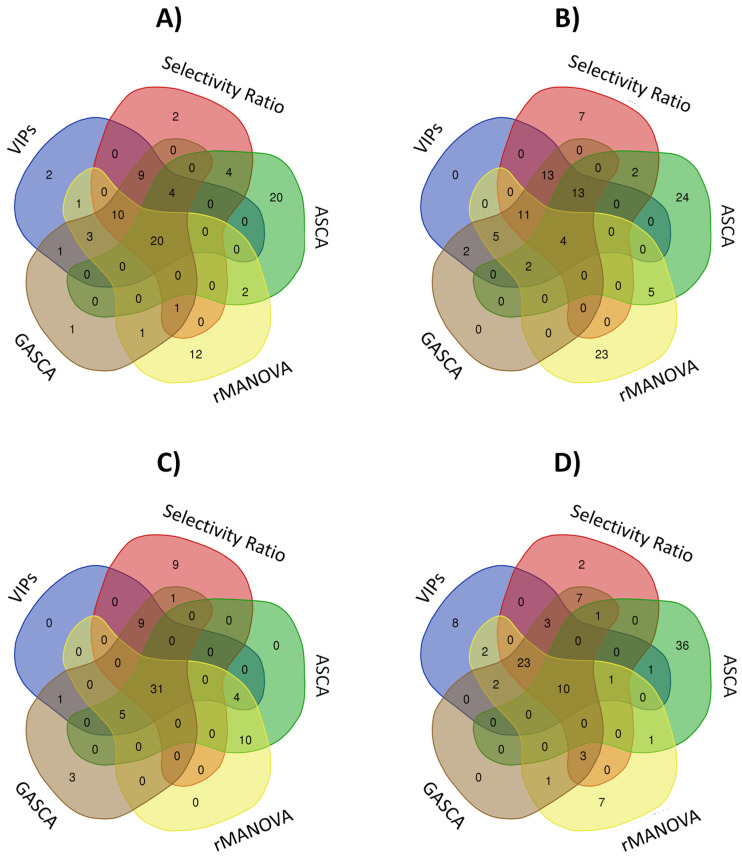
Venn diagrams summarizing the relationships among the 50 different selected variables detected for each data set. (**A**) TICs matrix for yeast positive MS ionization mode; (**B**) Features matrix for yeast positive MS ionization mode; (**C**) Zebrafish embryos exposed to low–dose BPA; and (**D**) Zebrafish embryos exposed to high–dose BPA.

Next, the matching variables selected as relevant from the different approaches were compared, taking as a benchmark the variables determined by the field’s reference (i.e., PLS-DA VIP Scores). Figure 3 shows the logical relations between these selected relevant variables for the different approaches using Venn diagrams. Here, four cases were considered: TICs and features matrices of the yeast study in the positive mode and at two levels of BPA exposure to zebrafish embryos. In all cases, the total number of relevant variables has been limited to 50 to focus on the variables with greater importance, and in an attempt to avoid coincidences by chance. In the case of PLS-DA VIP scores and Selectivity Ratio methods, those 50 variables with the higher values were selected. For the ANOVA-based methods, these variables showing the 50 largest loadings values in absolute value for the first component were selected. As shown above, this first component was enough to distinguish between the various sample types for the studied cases when considering the related factor matrix from the ANOVA decomposition. The results obtained in the analysis of the data from the rest of the examples are shown in the Supplementary Materials, giving concordant results (Figure S2).

Figure 3A shows results from the study using the TICs obtained for yeast in positive ionization mode. The Venn diagram showed that 20 variables are common to all considered approaches. Only ASCA (20) and rMANOVA (12) presented a relevant number of unique variables detected only by one method. These results confirmed the previous evidence in which the variable selection profiles or loadings associated with each method were evaluated. GASCA was the ANOVA method that provided the most similar results compared with PLS-DA. Figure 3B shows the evaluation of the corresponding selected variables obtained after preprocessing the LC-MS yeast samples in the positive mode. In this case, the number of variables common to all approaches is much lower (4). Again, only ASCA and rMANOVA have many unique variables (causing this low number of coincident variables). When considering the study of zebrafish embryos treated with BPA at two dose levels (Figure 3C,D), the obtained results led to similar conclusions. However, ASCA showed a different behaviour compared to all the other methods. For instance, in the control vs. high BPA exposure study, ASCA had many unique variables that avoided the coincidence from other methods. A list of identified metabolites present in zebrafish embryos is included in Table S5 (only the compounds that were characterized at MS/MS level are included). The significance obtained with each of the methods tested (VIPs, selectivity ratio, GASCA, ASCA, and rMANOVA) is included for each compound. Again, ASCA provided higher statistical values to different compounds than the rest of the methods, in agreement with the analysis from Figure 3.

**Figure 4:**
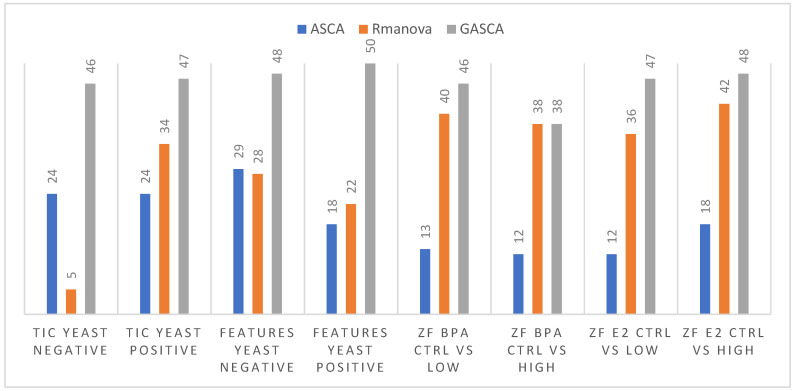
Comparison of the number of coincident variables detected by PLS–DA and the considered ANOVA–based methods. Maximum possible number of coincidences is 50.

Finally, the univariate ANOVA and multivariate ASCA-based profiles were individually compared with those retrieved by the PLS-DA VIP-Scores approach (Supplementary materials Table S2). In the case of yeast TICs data, the coincidence of the detected variables with PLS-DA was larger with GASCA (47 of 50), followed by rMANOVA (34), and finally ASCA (24 of 50). When comparing the selected variables with the PLS-DA, this trend occurred in most studied cases (Figure 4). In summary, rMANOVA and GASCA could be better options if the main goal of the study is variable selection after the ANOVA decomposition stage. This fact confirmed the theoretical basis from which the ASCA method had the initial purpose of statistical assessment of factors in experimental design and data exploration by SCA. However, the newly developed methods such as rMANOVA or GASCA showed advantages when the aim of the study was to perform feature detection to characterize the experimental design factors.

## 3. Materials and Methods

### 3.1. Chemicals and Reagents

Bisphenol A (BPA, ≥99.0%), 17-β-estradiol (E2, ≥98.0%) methylene blue (certified by the Biological Stain Commission, ≥82.0%), calcium sulphate (CaSO_4_·2H_2_O, ≥99.0%), dimethyl sulfoxide (DMSO, for molecular biology, ≥99.9%), potassium hydroxide (KOH, ≥85.0%), ammonium acetate (NH_4_Ac, ≥99.0%), formic acid (HForm, ≥95.0%), acetic acid (HAc, ≥95.0%), phosphate buffered saline (PBS), yeast extract, bacteriological peptone, and D-glucose were purchased from Sigma-Aldrich (Merck, Darmstadt, Germany). Ammonium formate (NH_4_Form, ≥99%) was obtained from Fluka Analytical (Honeywell, Muskegon, MI, USA). Chloroform (CHCl_3_, ≥99.0%) was provided by Carlo-Erba reagents (Dasti Group, Milan, Italy). Instant Ocean sea salt was purchased from Aquarium Systems (Sarrebourg, France), whereas dried flakes were obtained from TetraMin (Tetra, Melle, Germany). HPLC grade water and acetonitrile (AcN) were supplied by Merck KGaA (Merck, Darmstadt, Germany), and methanol (MeOH) HPLC grade from Fisher Chemical (Thermo Fisher Scientific, Fair Lawn, NJ, USA). L-methionine sulfone was purchased from Sigma-Aldrich (St. Louis, MI, USA). Lipid standards used were purchased from Avanti Polar Lipids (Alabaster, AL, US). The glycerophospholipids and triacylglycerides (PL) standards mix included: 1,2,3-17:0 triglyceride (TG), 1,3-17:0 (d5) diglyceride (DG), 17:0 cholesteryl ester (CE), 16:0 D31-18:1 phosphatidylethanolamine (PE), 16:0 D31-18:1 phosphatidylserine (PS), 16:0 D31-18:1 phosphatidylglycerol (PG), 16:0 D31-18:1 phosphatidylcholine (PC), 17:1 lyso PC (LPC), 17:1 lyso PE (LPE), 17:1 lyso PG (LPG), and 17:1 lyso PS (LPS). The sphigolipids (SL) standards mix included: N-dodecanoylsphingosine, N-dodecanoylglucosyl-sphingosine, and N-dodecanoylsphingosylphosphorylcholine.

### 3.2. Yeast Experiments

First, the performance of the different statistical methods was explored by studying the growth of yeast culture and considering the method used for lipid extraction (either for glycerophospholipids or sphingolipids) as an experimental factor. Therefore, results obtained from the two lipid types of extractions are compared.

#### 3.2.1. Culture Growth

A preculture of *Saccharomyces cerevisiae* (BY4741 strain) was kept at 30 °C and agitated at 150 rpm in yeast extract peptone dextrose (YPD) medium (composed of 20 g·L^−1^ bacteriological peptone, 10 g·L^−1^ yeast extract, 20 g·L^−1^ glucose at 40%) for 48 h [25]. Inoculation with the preculture was performed with a fresh YPD medium to an absorbance of 0.1 at 600 nm (A_600_). When an A_600_ of 0.6 was reached, 20 mL of each culture were taken, including five biological replicates for each condition. Fractions were centrifuged (3 min at 3000 rpm at 4 °C) and washed twice with PBS. The supernatant was discarded, and pellets transferred to Eppendorf tubes were kept at −80 °C until extraction.

#### 3.2.2. Lipid Extractions

Lipids from yeast samples were extracted by two different procedures, based on the previous work from Puig-Castellví [25] and Dalmau [26], with minor modifications. The first approach was a general lipid extraction, mainly targeting glycerophospholipids and triacylglycerides (Extraction A). The second extraction included a saponification step focused on the analysis of sphingolipids (Extraction B).

Extraction A started with the addition of 400 μL of Milli-Q water to the frozen samples. Then, samples were vortexed and transferred to glass vials. Next, 1 mL of MeOH, 2 mL of CHCl_3_, and 40 μL of PL standard mix at a concentration of 20 μM were added. Vortex and ultrasonication steps were applied to the vials in cycles of 3 min and 15 min, respectively. Glass beads were added, and the previous step was repeated twice. Samples were left overnight at 48 °C in a thermostatic bath, then evaporated to dryness under nitrogen gas and stored at −80 °C until use. Before analysis, extracts were re-suspended in 800 μL of MeOH, centrifuged 3 min at 10,000 rpm at 4 °C, and aliquots of 200 μL were transferred to chromatographic vials, where a 10 μL aliquote of SL standards mix was added to each vial.

Extraction B differed in the initial proportion of the employed MeOH/CHCl_3_ mixture, which was 2/1 in this case. The SL standard mix was added at the beginning in the same proportion as PL for set A. After overnight incubation, 75 μL of KOH of 1 M in MeOH were added to the samples, that were then sonicated for 15 min and kept for 2 h at 37 °C (i.e., a saponification step). Next, KOH was neutralized by adding 75 μL of 1 M HAc, and samples were evaporated until dryness under nitrogen gas and stored at −80 °C until further use. Before analysis, samples were resuspended as for set A, adding 10 μL of PL standards mix instead of the SL standards mix.

Quality controls samples (QCs) were composed of 25 μL of each biological replicate from each set of samples (extracted samples A and B).

#### 3.2.3. LC-MS Analysis

A total of five samples (biological replicates) of each extraction were randomly analyzed; one sample per set of extraction was also analyzed in triplicate (instrumental replicates). In total, 16 chromatograms were obtained (eight for each extraction batch). QCs and blanks were also interspersed in the chromatographic sequence. LC-MS analysis was carried out with a Waters Acquity UPLC system coupled to a Waters LCT Premier orthogonal accelerated time-of-flight mass spectrometer (Waters), operated in both positive (ESI+) and negative (ESI-) electrospray ionization modes. Full-scan spectra from 50 to 1500 Da were acquired at a scan cycle time of 0.3 s. The chromatographic method employed was described previously [25]. Briefly, an RP C8 Acquity UPLC bridged ethylene hybrid (Waters) column of 100 mm × 2.1 mm i.d. (1.7 μm) was employed. Mobile phases were: (A) MeOH 1 mM NH_4_Form and 0.2% HForm; (B) H_2_O 2 mM NH_4_Form and 0.2% HForm. The solvent elution gradient started at 80%A, increased until 90%A at 3 min, held at 90%A until minute 6 min, increased to 99%A at 15 min, and held at 99% until 18 min. Then, the column was re-equilibrated during 2 min. Flow rate, injection volume and column temperature were set at 0.3 mL min^−1^, 10 μL and 30 °C, respectively.

### 3.3. Zebrafish Embryos Experiments

The second dataset aimed to evaluate of the effects caused by two endocrine-disrupting chemicals (BPA and E2) in zebrafish embryos. In this study, we assessed the impact of the different concentration exposure levels in the development of zebrafish embryos by considering the changes in the metabolome.

#### 3.3.1. Zebrafish Maintenance

Adult wild-type zebrafish (*Danio rerio*) were fed twice a day with dried flakes and maintained at a temperature of 28 ± 1 °C, with photoperiods (light-night) of 12 h. Fish water, prepared in Milli-Q water, contained 90 μg·mL^−1^ of Instant Ocean sea salt and calcium sulphate (100 μg·mL^−1^), as previously reported [27]. Zebrafish embryos were obtained by natural mating placing five females and three males in 4-L breeding tanks. Eggs were separated from adults through a bottom mesh. At 2 h post-fertilization (hpf), eggs were collected and rinsed. At 24 hpf, fertilized eggs were washed three times with 0.0002% methylene blue and randomly distributed in 6-well multiplates as follows: 15 individuals per 5.0 mL of fish water, 8 replicates of each condition, in different plates, to account for possible “tank” effects.

All experiments were approved by the Institutional Animal Care and Use Committees at the Research and Development Centre of the Spanish National Research Council (CID-CSIC) and were also conducted under the institutional guidelines under a license from the local government (DAMM 7669, 7964).

#### 3.3.2. Exposure Protocols

BPA and E2 working solutions were prepared daily in fish water at a final concentration of 0.2% DMSO by diluting from stock solutions at higher concentrations in DMSO, previously prepared and kept at 4 °C until use. Exposure concentrations were chosen by a preliminary range-finding test and based on previous studies [27,28].

Until 48 hpf, embryos were kept in fish water to avoid early embryonic processes. Then, exposure started, and solutions were changed daily to ensure continuous exposure to the contaminant until embryo collection at 120 hpf. Control samples in 0.2% DMSO (without treatment) were also included in the multiplates. The following concentrations were used as low and high nominal exposure concentrations and using DMSO as a vehicle; BPA concentration levels were set to 4.4 and 17.5 μM, respectively, whereas, for E2, 1 and 4 μM concentrations were used, respectively. Pools of 30 zebrafish embryos were gathered (15 + 15 from different wells from the same plate) for each biological replicate. A total number of three biological replicates per treatment were used for LC-MS.

#### 3.3.3. Metabolite Extraction

The frozen Eppendorf tubes containing the embryos were kept in dry ice. Then, 0.900 mL of methanol and 90 μL of L-methionine sulfone at 50 mg L^−1^ were added to each sample. Samples were vortexed, sonicated for 15 min, and centrifuged at 14,500 rpm for 10 min at 4 °C. Next, the supernatant was isolated, and 500 μL of water and 300 μL of CHCl_3_ were added. Samples were vortexed again, placed on ice at 4 °C, and centrifuged under the same conditions. Aqueous fractions (upper layer) were collected and evaporated to dryness under nitrogen gas. Samples were re-suspended in 100 μL of AcN:H_2_O (1:1), centrifuged, and transferred to a chromatographic vial, where they were evaporated until dryness and kept at −80 °C. Finally, extracts were re-suspended before injection with 100 μL of AcN:H_2_O (1:1).

Quality control (QC) samples were generated by pooling 10 μL of an extract from each condition studied (two concentration levels of both compounds, BPA and E2, plus control samples).

#### 3.3.4. LC-MS Analysis

Three biological replicates were analyzed for each sample condition (control, low, and high exposure concentrations) and each treatment (BPA or E2). In total, 18 samples were randomly analyzed, with QCs and blanks added in the sequence.

Chromatographic separation was carried on a 1290 Infinity II HPLC system (Agilent Technologies, Santa Clara, CA, USA), using a HILIC column (TSK Gel Amide-80 column: 250×, 2.1 mm; 5 μm) from Tosoh Bioscience (Tokyo, Japan) at room temperature. The chromatographic method was adapted from a previous work [27]. Briefly, mobile phases composition were: (A) 5 mM of NH_4_Ac adjusted to pH 5.5 with HAc, and (B) AcN. The solvent elution gradient started at 25% of A, increased to 30% of A at 8 min, then to 60% A at 10 min, and held until 12 min. Then, the column was re-equilibrated for 8 min. The flow rate was set at 0.15 mL min^−1^, the injection volume was 5 μL, and the autosampler temperature was 4 °C.

A 6545XT AdvanceBio LC/Q-TOF (Agilent Technologies, Santa Clara, CA, USA) with a Dual AJS ESI source was employed in negative ionization mode. High-resolution mass spectrometry conditions were set as follows: gas temperature, 250 °C; drying gas, 13 L min^−1^; nebulizer, 35 psi; shealth gas temperature and flow, 350 °C and 12 L min^−1^, respectively. Mass range was set from 50 to 1700 Da, with an acquisition frequency of 333.33 ms/spectrum. An auto MS/MS protocol was set for obtaining iterative MS/MS fragmentations of the QCs and collision energy was set to 20 eV.

### 3.4. Data Analysis

**Figure 5:**
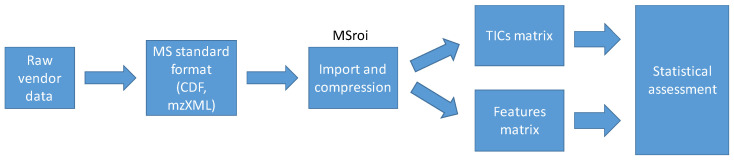
Workflow of the data analysis strategy from the MS raw data acquisition to the statistical assessment.

Figure 5 summarizes the main steps of the strategy followed to analyze the LC-MS data sets and is described in detail in the subsections below.

#### 3.4.1. Data Import and Compression

First, LC-MS raw data acquired using the vendor software was transformed into MS open data formats (the first step in the workflow from Figure 5). Waters LC-MS chromatograms (.raw) from yeast samples were transformed into the CDF format using the Databridge function (MassLynx 4.1 software, Waters, Milford, MA, USA). However, Agilent LC-HRMS chromatograms (.d) from zebrafish embryos exposure study were transformed into the mzXML format in centroid mode using the MSConvert tool from the ProteoWizard suite (64-bit, 3.0.20361 version) [29].

The next step consisted of importing these files into the selected computing platform (MATLAB, Release 2020b, The Mathworks Inc., Natick, MA, USA). Here, total ion current (TIC) chromatograms were directly obtained. In addition, a features matrix containing only those signals with intensity over a pre-defined threshold was also generated. In this work (the second step of Figure 5), the MSROI approach was applied to perform this data importing procedure and, simultaneously, spectral compression [30,31]. Regions of interest (ROI) parameters used in each case are shown in Table S3.

After this procedure, the matrices to be analyzed were built up (third step of Figure 5). On the one hand, the TIC chromatogram for every sample allowed to build up a matrix including all TIC information (size of this matrix was the number of samples by the number of points in the time axis, i.e., retention times). On the other hand, the MSROI procedure generated a features matrix containing the peak areas of the detected features (defined by a *m*/*z* value) for each sample [32].

Then, these TIC chromatograms and feature matrices were independently normalized to correct the instrumental intensity drifts among injections. This normalization procedure was performed by dividing all the variables’ areas (by sample) by the mean area of surrogates and internal standards for each sample (SL and PL lipid standards mixture for the yeast and L-methionine for the zebrafish embryos studies) and the amount of sample considered (A_600_ values for yeast and number of embryos for zebrafish studies).

#### 3.4.2. Statistical Assessment

The statistical evaluation of the TIC and features matrices followed a common workflow (the last step of the workflow depicted in Figure 5).

First, principal component analysis (PCA) was applied to perform a preliminary data exploration. PCA scores enabled a visual comparison of the samples according to the experimental design employed in each study (i.e., the family of lipids considered in the case of yeast samples and exposure level in the case of zebrafish embryo samples) and detection of potential outliers. In addition, the evaluation of PCA loadings can also provide preliminary insights regarding the variables more related to a particular sample type. However, in general, the determination of these variables is somewhat arbitrary and analyst-dependent. PCA was applied to mean-centered (TICs) and autoscaled (features) matrices.

Next, different approaches were tested to identify the most relevant features linked to the experimental design. In the omics field (and, in particular, metabolomics), the determination of these most relevant features (i.e., potential biomarkers) has been widely carried out using univariate techniques based on statistical hypothesis testing. Depending on the experimental design (i.e., number of groups) and the properties of the data, parametric (i.e., Student’s *t* and univariate ANOVA) or non-parametric tests (i.e., Wilcoxon test or Kruskal-Wallis) are used. However, when many features are considered, multiple hypotheses testing can lead to an uncontrolled number of false-positives [33]. To overcome this problem, different approaches have been proposed to minimize the number of false-positives in the selection of these potential markers. Here, *t*-tests were performed for binary (two types of samples) comparison whereas ANOVA tests were employed for studies involving ternary comparisons. The list of variables selected by these statistical hypothesis approaches was corrected in this work by the Benjamini-Hochberg procedure [34]. Only these variables with a corrected *p*-value lower than 0.05 were considered statistically significant.

An alternative to multiple hypothesis tests (i.e., one test for each feature) is to adopt a multivariate approach. The standard approach in MS-based metabolomics is the application of partial least squares discriminant analysis (PLS-DA). The most relevant variables were identified from the generated model using approaches such as the selectivity ratio (SR) [8] or VIP scores [9]. SR method is based on calculating the ratio between explained and unexplained variances for each variable in the target projection vector. This approach combines the regression vector and the variance/covariance of the data matrix to identify which variables are more relevant in the classification model. In contrast, VIP scores are calculated as the weighted sum of the squares of the PLS weights relating each latent variable with the amount of explained variance for the correct class classification. Therefore, variables with a large VIP score were associated with a better description of the class belonging. Usually, variables with a VIP score greater than one are selected as relevant, considering that the average of the squared VIP scores equals one. However, in the literature, several papers describe the benefits of this approach, as well as its potential limitations [5,11,35]. In this work, PLS-DA models were built on the mean-centered total ion current chromatograms (TICs) and autoscaled for features (i.e., defined by a particular *m*/*z* value from the MSROI approach) matrices. The reliability of the obtained features was assessed by means of the calculation of 1000 replicate PLS-DA models, randomly removing between 1% and 10% of the total number of variables as described in Deng et al. [36]. Selected variables after VIP scores or selectivity ratio determination were almost the same for the different considered conditions (see Supplementary Materials for more details).

Since many omic studies are based on statistically designed experiments, several chemometric methods have been proposed in recent years to extract the statistically relevant information related to the factors used in the experimental design. Here, three different approaches were evaluated.

First, ASCA analysis was applied to statistically assess the significance of the design factors used in both studies and to determine the most relevant variables associated with these factors. ASCA combines the variance decomposition power of ANOVA according to the experimental design, with the ability to explore the effects caused for all variables through Simultaneous Component Analysis (SCA) [37]. This analysis strategy enables independent evaluation of the statistical significance of each experimental factor (and possible factor interactions). It is recommended that ASCA is applied to well-balanced sample designs [15,19]. Only in this case, the sum of squares (SSQ) of elements of the ANOVA decomposed matrices represents appropriately the amount of variance of the original matrix explained by each factor and by their interaction. When the experimental design is unbalanced, corrections for the calculation of these sums of squares are required to define the type II SSQ and type III SSQ. Next, the statistical significance of each factor (and of their interaction) is estimated by means of a permutation test, evaluating the null hypothesis H_0_ (no experimental effect) against the alternative hypothesis H_1_ (experimental effect). This test is performed by calculating the SSQ of the data in the considered matrix and of the SSQ values obtained when rows of the matrix are permuted [15]. A *p*-value was then calculated by considering the number of permuted SSQ values larger than the original SSQ and the total number of permutations performed. In addition, the evaluation of SCA scores and loadings provide information regarding sample and variable distribution and the importance for each considered factor. The ASCA loadings obtained for each factor show the more relevant variables for its modelling. Here, TICs and features’ area matrices were mean-centered before ASCA analysis, and the number of iterations for the permutation test was set to 10,000.

The assumption of non-correlation between variables means that ASCA might not be a reliable option for feature detection in metabolomics studies, since the behavior of some of the studied variables (metabolite concentrations) might be correlated.

Next, rMANOVA was used to evaluate TIC and features data matrices for both studies. This method proposed by Engel in 2015 [21] overcomes the limitations of sample size (MANOVA) and the correlation between variables (ASCA). The critical step of rMANOVA is determining the optimal regularization factor (δ, in a range between 0 and 1) that is calculated according to the Ledoit-Wolf theorem [38]. Depending on the value of this regularization factor, the rMANOVA model will be equal to a MANOVA model (δ = 0) or to an ASCA model (δ = 1). However, the most common situation is that this factor adopts intermediate values in which the advantages of rMANOVA models are more relevant. Finally, the statistical assessment of the experimental factors is performed using a permutation test, as described above for ASCA. However, compared with ASCA, in some circumstances, rMANOVA can allow more straightforward determination of the most relevant features. Engel’s implementation of the rMANOVA algorithm has been used in this work. TICs and features matrices were mean-centered before the analysis, and the number of permutations for the permutation test was set to 10,000.

Finally, the last method used in this work is group-wise ANOVA simultaneous component analysis (GASCA) proposed by Saccenti [22]. GASCA attempts to overcome some ASCA limitations by applying the group-wise PCA (GPCA) [39] in the second step after ANOVA decomposition. The GPCA algorithm relies on the sparsity of loadings to increase the simplicity and interpretation of the generated model by considering relationships between variables (metabolites). Due to the impact of the GPCA model on the obtained loadings for each factor, the potential usefulness of this approach for feature detection should be tested. As in the previous cases, balanced experimental designs are preferred to simplify the analysis, and the statistical assessment is performed through a permutation test (10,000 permutations used). Data were mean-centered before the analysis.

#### 3.4.3. Software Used

Univariate statistical tests were performed by using *t*-test and anova1 functions available at the MATLAB Statistics and Machine Learning Toolbox (MATLAB 2020b, The Mathworks Inc, Natick, MA, USA). Obtained *p*-values were adjusted by the Benjamini-Hochberg algorithm available at the FalseDiscovery library published at the github.com/carbocation/falsediscovery (accessed on 9 May 2022). ANOVA PLS-DA and ASCA were performed using PLS Toolbox 8.9.1 (Eigenvector Research Inc, Wenatchee, WA, USA), working under MATLAB 2020b. The MATLAB source code of the regularized MANOVA is available at the following github repository: github.com/JasperE/regularized-MANOVA (accessed on 9 May 2022). The GASCA algorithm is also freely available in the MATLAB MEDA toolbox and can be downloaded from the address: github.com/josecamachop/MEDA-Toolbox (accessed on 9 May 2022). Venn diagrams were generated using the tool from the Bioinformatics & Evolutionary Genomics group at VIB/UGent (bioinformatics.psb.ugent.be/webtools/Venn/, accessed on 9 May 2022).

#### 3.4.4. Metabolite Identification

Metabolites in zebrafish QC samples were identified based on the MS/MS spectral matches using public metabolite libraries from the MS-DIAL website [40]. The parameters employed for MS-DIAL software are included in Table S4. The identified compounds, their significance, and other relevant information (e.g., HMDB code, chemical formula, retention time) are included in Table S5.

## 4. Conclusions

In this work, we have evaluated the ability of three multivariate ANOVA-based methods to determine the statistical significance of the experimental design factors (e.g., lipid extraction protocol, pollutants dose of exposure) and their ability to select relevant variables linked to these factors.

On the one hand, the evaluation of the statistical assessment indicated that ASCA determined the statistical significance where it was expected to exist based on the previous biological knowledge of the experiment and its experimental design. In contrast, GASCA provided some inconsistent results as, in some cases, factors were not pointed as statistically significant when they were expected to be. One possibility to improve these statistical significance results and the interpretation of multivariate ANOVA-based methods could be the use of resolved elution profiles (or areas derived from them) of the different sample constituents resolved by chemometric methods, such as MCR-ALS.

On the other hand, GASCA was the ANOVA-based method that provided a list of relevant variables most similar to the variable list provided, considering the VIP scores obtained by the PLS-DA method. In addition, this variable selection step was the major weakness of ASCA since the obtained variables list was the most dissimilar when compared to variables pointed by all the other methods.

In both cases (i.e., considering the statistical significance and variable selection), rMANOVA showed acceptable results. Therefore, rMANOVA could be an option if both statistical assessment and feature detection studies are performed. In contrast, ASCA and GASCA could be employed for only statistical assessment or variable selection, respectively. Table 2 summarizes the main advantages and limitations of each multivariate ANOVA-based method, as well as gives some recommendations regarding the use of each method. In addition, a more comprehensive study to elucidate the impact of the different methods will require an experimental design with a larger number of samples to reinforce the obtained conclusions. Finally, it should be noted that the results obtained for each method are dataset-dependent and, despite that the main trends should be conserved, different results regarding the statistical significance or variable selections could be obtained depending on the data structure.

## Figures and Tables

**Table 1:** Summary of the statistical assessment study for the considered datasets showing obtained *p*-values for the different ANOVA-based approaches.

Dataset		Experimental Factor	ASCA	rMANOVA	GASCA
TIC Matrix	Yeast–MS negative ionization mode	Type of lipid extraction	0.0001 (0.0007 *)	0.0001	0.039 *
	Yeast–MS positive ionization mode	Type of lipid extraction	0.0001 (0.0001 *)	0.0001	0.001 *
Features Matrix	Yeast–MS negative ionization mode	Type of lipid extraction	0.0001 (0.0001 *)	0.0001	0.002 *
	Yeast–MS positive ionization mode	Type of lipid extraction	0.0001 (0.0001 *)	0.0001	0.002 *
	Zebrafish embryos–BPA exposure	Exposure concentration			
		Control vs. Low	0.0001	0.0001	0.09
		Control vs. High	0.0001	0.0001	0.10
		Control vs. Low vs. High	0.0001	0.0001	0.01
	Zebrafish embryos–E2 exposure	Exposure concentration			
		Control vs. Low	0.4472	0.0001	0.47
		Control vs. High	0.0001	0.0001	0.22
		Control vs. Low vs. High	0.0093	0.0001	0.35

* Balanced data (a sample was eliminated from the set).

**Table 2:** Summary of the main advantages, limitations, and opportunities of the considered ANOVA-based methods.

	ASCA	rMANOVA	GASCA
**Advantages**	Widespread use in metabolomics (reference multivariate statistical method) Best match between experimental and expected significance	Best of both worlds (model depending on data I MANOVA and ASCA)	A good option for sparse data (i.e., metabolomic datasets) Best match with VIPs from PLS-DA for identifying significant variables
**Limitations**	Most dissimilar matches identifying significant variables compared to VIPs from PLS-DA It assumes metabolites are not correlated and that they all have the same variance.	Dissimilar matches with VIPs from PLS-DA in selection of relevant variables	Very strict for determination of significant factors (only factors with very low *p*-values in other methods will appear as significant)
**Opportunities**	Good choice when combined with PLS-DA (VIPs) for the determination of the significant variables	Good choice when aiming one method for statistical analysis and selecting relevant variables (but further validation on the variables is desirable)	Good option for assessing the significance of variables and factors when big effects are encountered (very significant factors in the DOE)

## Data Availability

The data presented in this study are openly available in Zenodo (https://zenodo.org/record/6384813, accessed 8 May 2022).

## Supplementary Materials

The following supporting information can be downloaded at: www.mdpi.com/article/10.3390/molecules27103304/s1; Table S1. Comparison of profiles obtained for variable selection. Values are the correlation coefficient of the absolute values of the vector profiles. Table S2. Logical relationships between the features detected by PLS-DA and FDR-corrected statistical tests, Selectivity ratio, ASCA, rMANOVA and GASCA. Shadowed columns represent common features between the two compared methods and Bold characters highlights those comparison with a number of coincidences higher than 80%. Table S3. ROI parameters selected for each dataset. Table S4. Parameters employed for MS-DIAL analysis. Table S5. Tentative identification of metabolites of endocrine disruption on zebrafish embryos and their significance with the different statistical methods. Figure S1. Zebrafish embryos exposed to a low-dose of estradiol. PCA analysis: PC1 vs. PC2 scores plot. Figure S2. Venn diagrams summarizing the relationships on the variables detected for each data set. (A) TICs matrix for yeast negative; (B) Features matrix for yeast negative; (C) Zebrafish embryos exposed to low-dose estradiol; and (D) Zebrafish embryos exposed to high-dose estradiol.
