# Supplementary material for: Comparison of Multivariate ANOVA-Based Approaches for the Determination of Relevant Variables in Experimentally Designed Metabolomic Studies
Source: Molecules. 2022 May 20;27(10):3304. doi: 10.3390/molecules27103304 (PMC9147242; doi:10.3390/molecules27103304)

Supplementary Material

Comparison of multivariate ANOVA-based approaches for the determination of relevant variables in experimentally designed metabolomic studies

Miriam Pérez-Cova[1,2], Stefan Platikanov[1], Dwight R. Stoll[3], Romà Tauler[1] and Joaquim Jaumot[1,*]

[1] Department of Environmental Chemistry, IDAEA-CSIC, Jordi Girona 18-26, E08034 Barcelona, Spain
[2] Department of Chemical Engineering and Analytical Chemistry, University of Barcelona, Diagonal 647, Barcelona, E08028, Barcelona, Spain
[3] Department of Chemistry, Gustavus Adolphus College, 800 West College Avenue, Saint Peter, MN 56082, United States

Contents

A. Evaluation of the performance of PLS-DA models for feature selection.

Table S1. Comparison of profiles obtained for variable selection. Values are the correlation coefficient of the absolute values of the vector profiles.

Table S2. Logical relationships between the features detected by PLS-DA and FDR-corrected statistical tests, Selectivity ratio, ASCA, rMANOVA and GASCA. Shadowed columns represent common features between the two compared methods and Bold characters highlights those comparison with a number of coincidences higher than 80%.

Table S3. ROI parameters selected for each dataset.

Table S4. Parameters employed for MS-DIAL analysis.

Table S5. Tentative identification of me-tabolites of endocrine disruption on zebrafish embryos and their significance with the different statistical methods.

Figure S1. Zebrafish embryos exposed to a low-dose of estradiol. PCA analysis: PC1 vs PC2 scores plot

Figure S2. Venn diagrams summarizing the relationships on the variables detected for each data set. A) TICs matrix for yeast negative; B) Features matrix for yeast negative; C) Zebrafish embryos exposed to low-dose estradiol; and D) Zebrafish embryos exposed to high-dose estradiol.

A. Evaluation of the performance of PLS-DA models for feature selection.

We have calculated a large number of PLS-DA models removing a different number of variables according to these conditions:
- Datasets considered: 1) Yeast Tic Matrix positive ionization mode; and 2) Zebrafish embryos BPA exposure control vs high.
- Number of replications: 1000 (total number of PLS-DA models in each case).
- Number of variables eliminated in each model. We have tested three different levels: 1) 1% of the total variables; 2) 4% of the total variables; 3) 10% of the total variables.

Results show that the used feature selection process is reliable since the selected variables are practically identical. Below, a graphical representation of the obtained VIP scores is shown, focusing on the zebrafish dataset.

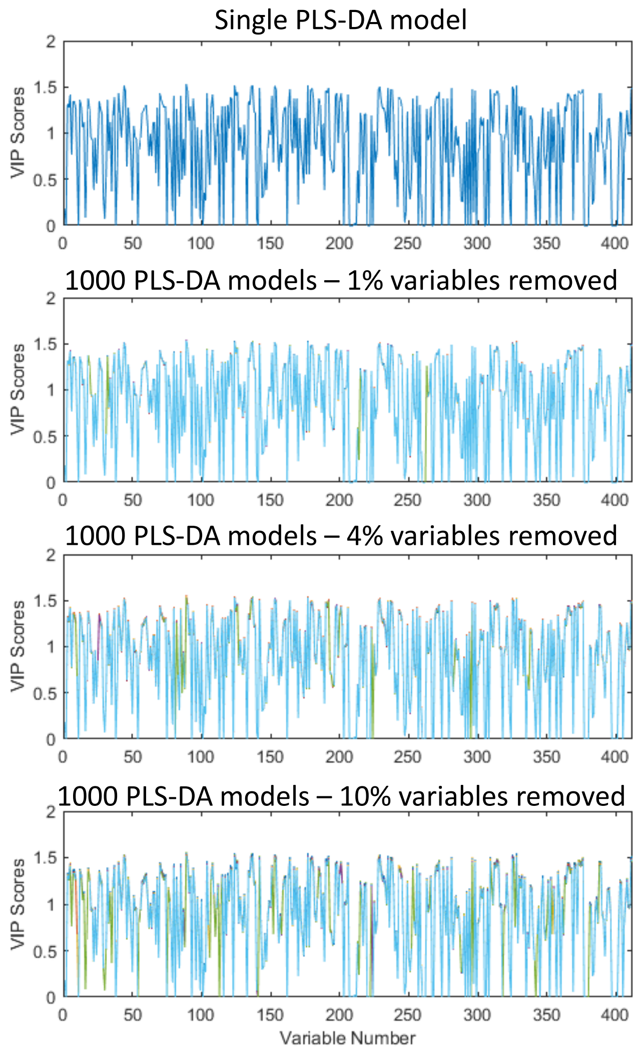

It can be seen that the obtained VIP scores profiles are almost identical despite that in the 1000 models of the bottom plot (10% of the samples removed in each permutation), subtle differences can be observed.

Analogous results were obtained when the selectivity ratio selected variables were considered. For this reason, we first evaluated the number of times that each variable is selected as one of the 50 more relevant VIP scores (considering that, especially in the last case, it will be removed several times from the analysis).

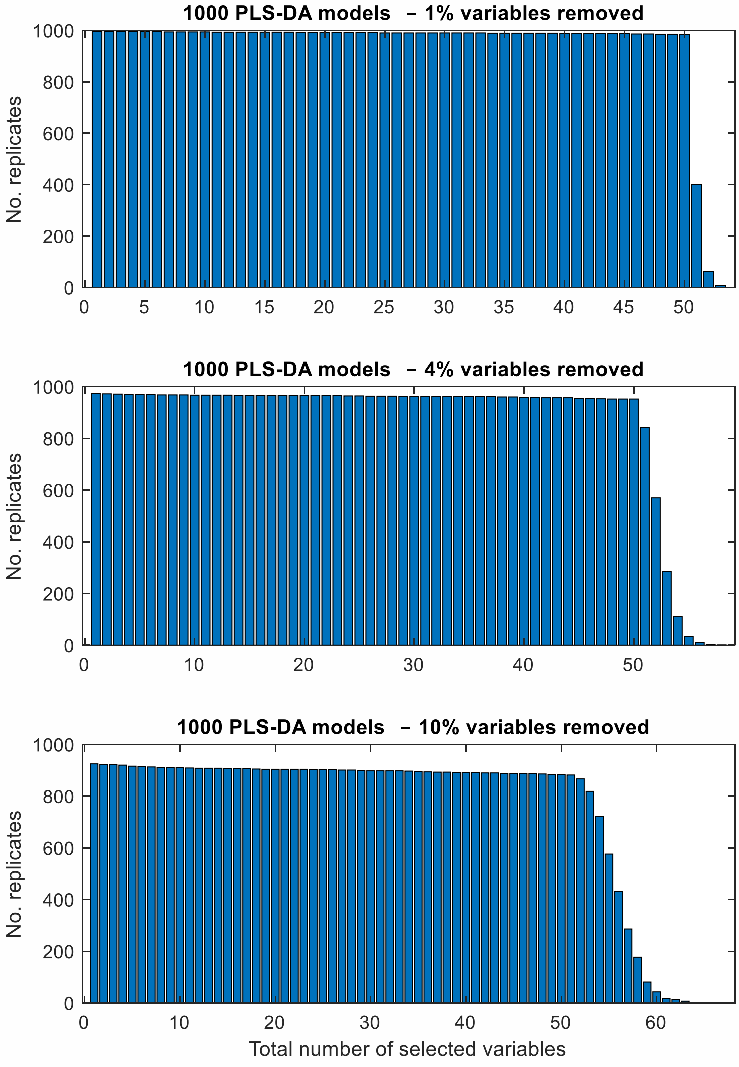

Finally, we represented a Venn diagram to evaluate the similarity between the variables determined in each case. It can be seen that 86% of the variables are detected in all cases, and if we consider the models calculated, removing less than 5% of the variables is 100%. Only, in the case of removing 10% of the variables, there are some non-coincident variables with the original model, although most of them are ranked between the 51 and 68 higher VIP scores.

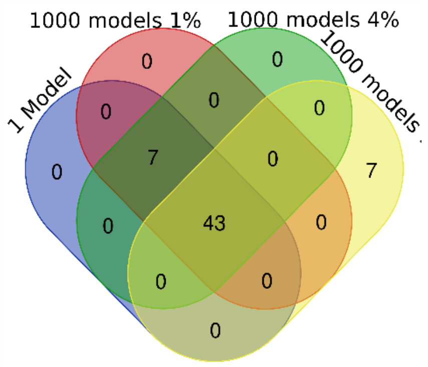

Table S1. Comparison of profiles obtained for variable selection. Values are the correlation coefficient of the absolute values of the vector profiles.

		VIPS	Selrat	ASCA	rMANOVA	GASCA
TIC Yeast negative	VIPS					
	Selrat	0.87				
	ASCA	0.80	0.86			
	rMANOVA	0.06	-0.05	-0.02		
	GASCA	0.89	0.84	0.76	0.04	
TIC yeast positive	VIPS					
	Selrat	0.55				
	ASCA	0.65	0.83			
	rMANOVA	0.32	0.56	0.41		
	GASCA	0.93	0.43	0.53	0.29	
Features yeast negative	VIPS					
	Selrat	0.90				
	ASCA	0.23	0.14			
	rMANOVA	0.54	0.31	0.18		
	GASCA	0.95	0.83	0.24	0.49	
Features yeast positive	VIPS					
	Selrat	0.57				
	ASCA	0.26	0.34			
	rMANOVA	0.30	0.35	0.21		
	GASCA	0.96	0.48	0.25	0.25	
Feature zebrafish BPA Ctrl vs Low	VIPS					
	Selrat	0.42				
	ASCA	0.28	0.26			
	rMANOVA	0.60	0.83	0.22		
	GASCA	0.95	0.33	0.26	0.48	
Feature zebrafish BPA Ctrl vs High	VIPS					
	Selrat	0.36				
	ASCA	0.28	0.08			
	rMANOVA	0.59	0.86	0.15		
	GASCA	0.94	0.29	0.27	0.47	
Feature zebrafish E2 Ctrl vs Low	VIPS					
	Selrat	0.76				
	ASCA	0.29	0.27			
	rMANOVA	0.83	0.61	0.23		
	GASCA	0.94	0.65	0.27	0.72	
Feature zebrafish E2 Ctrl vs High	VIPS					
	Selrat	0.70				
	ASCA	0.27	0.23			
	rMANOVA	0.88	0.82	0.24		
	GASCA	0.95	0.57	0.24	0.81	

Table S2. Logical relationships between the features detected by PLS-DA and FDR-corrected statistical tests, Selectivity ratio, ASCA, rMANOVA and GASCA. Shadowed columns represent common features between the two compared methods and Bold characters highlights those comparison with a number of coincidences higher than 80%.

	**FDR vs VIPS**			**Selectivity Ratio vs VIPs**		**ASCA vs VIPs**		**rMANOVA vs VIPS**		**GASCA vs VIPs**	
	FDR	Common	VIPs	SelRat VIPs	Common	ASCA VIPs	Common	rMANOVA VIPs	Common	GASCA VIPs	Common
TICs yeast negative	155	44	6	7	**43**	26	24	45	5	4	**46**
TICs yeast positive	411	50	0	7	**43**	26	24	16	34	3	**47**
Features yeast negative	3	19	11	5	**45**	21	29	22	28	2	**48**
Features yeast positive	342	50	0	9	**41**	32	18	28	22	0	**50**
Zebrafish BPA Ctrl vs Low	0	14	36	10	**40**	37	13	10	**40**	4	**46**
Zebrafish BPA Ctrl vs High	0	22	28	13	37	38	12	12	**48**	12	38
Zebrafish E2 Ctrl vs Low	0	2	48	13	36	38	12	14	36	3	**47**
Zebrafish E2 Ctrl vs High	0	0	50	4	**46**	34	16	8	**42**	2	**48**

Table S3. ROI parameters selected for each dataset.

Dataset	Signal-to-noise ratio threshold (%)	Min-max signal factor	Mass error tolerance	Minimum number of occurences	*m/z* values calculation
Yeast (ESI +)	0.4	4	30	70	Median
Yeast (ESI -)	1.4	2	30	10	Median
Zebrafish embryos (ESI -)	0.3	1	15	50	Median

Figure S1. Zebrafish embryos exposed to a low-dose of estradiol. PCA analysis: PC1 vs PC2 scores plot.

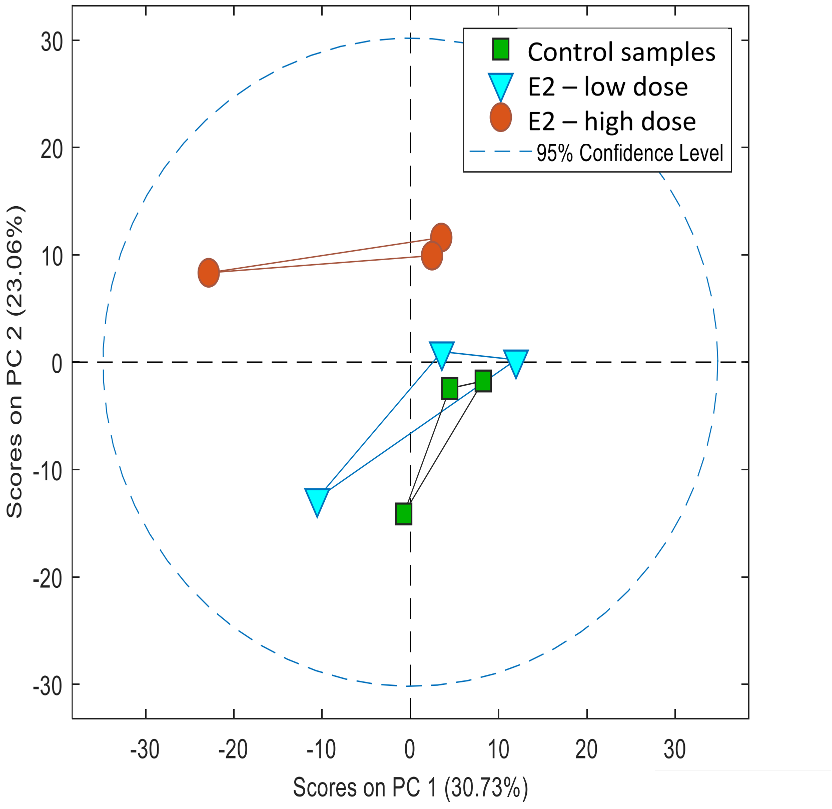

Figure S2. Venn diagrams summarizing the relationships on the variables detected for each data set. A) TICs matrix for yeast negative; B) Features matrix for yeast negative; C) Zebrafish embryos exposed to low-dose estradiol; and D) Zebrafish embryos exposed to high-dose estradiol.

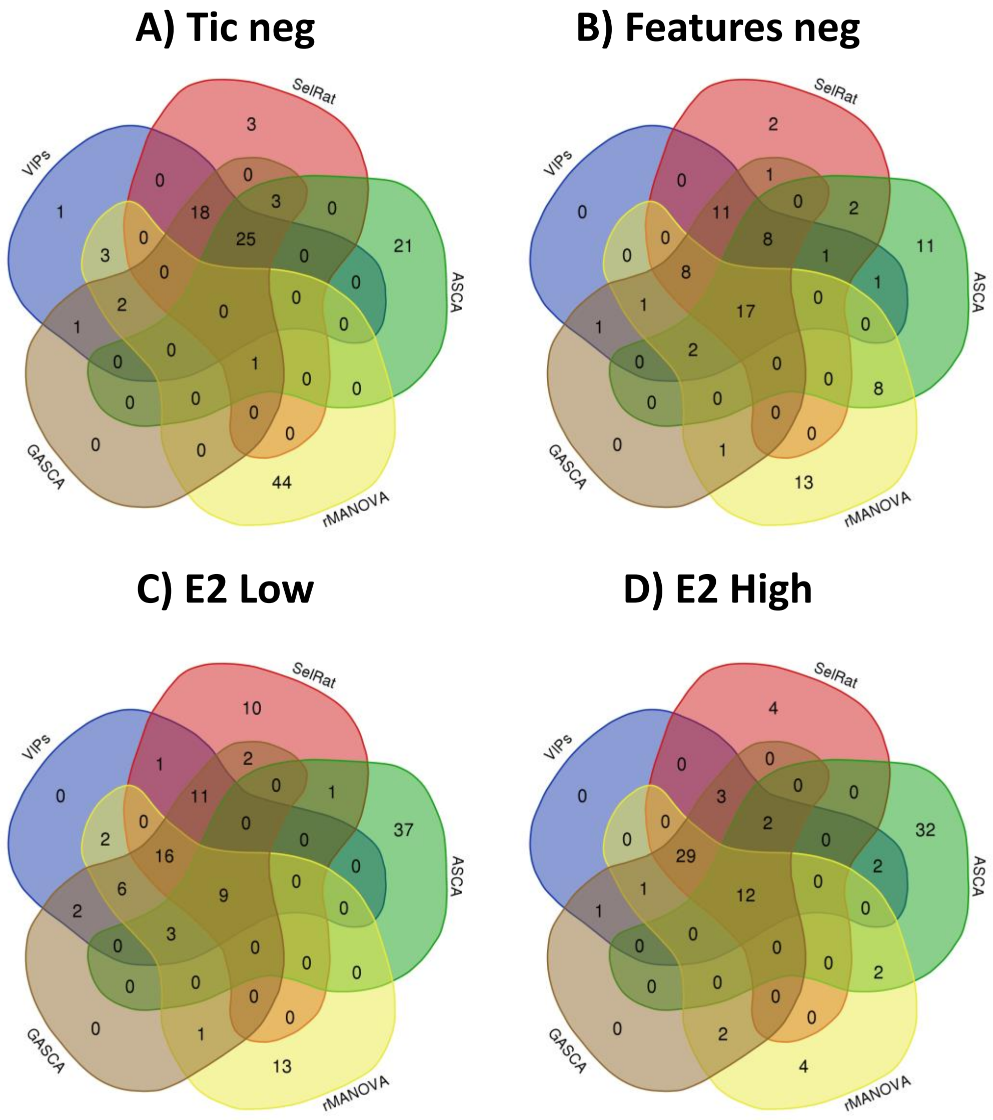

Supplement: Supplementary file 1 [file molecules-27-03304-s001.zip › SuppMaterialA_rev.pdf]
